# Addison's Anæmia

**Published:** 1902-05-31

**Authors:** 


### ADDISON'S ANÆMIA.

At the last meeting of the Pathological Society Dr. William Hunter made a communication dealing with the infective lesions of the tongue, stomach, and intestines in Addison's anæmia, the observations now recorded having been made on 25 well-marked cases of the disease which had come under his notice during the past two years. It is now some time since Dr. Hunter pointed out the connection between oral sepsis and pernicious anæmia, but he now goes a step further and would separate the infective agent by which Addison's disease is produced from the more ordinary septic infections by which certain forms of gastritis are set up. It would seem that there are first certain conditions of stomatitis and gingivitis which may be grouped together as conditions of oral sepsis; secondly, that as the result of long-continued oral sepsis there may be a real septic gastric catarrh; and, thirdly, in addition to this there may be a septic enteritis of similar origin. But these conditions by themselves, however severe they may be, are not sufficient to cause pernicious anæmia, although they constitute a most important etiological factor in the development of the disease, creating lesions of the mucosa in the alimentary canal favourable to any subsequent infection to which the tract may be exposed. This subsequent infection, this specific cause of the disease, is to be found in a peculiar form of glossitis which has been met with in every case that Dr. Hunter has seen since 1888—a glossitis of a special character depending on some deep-seated infection of the tongue itself. Thus in connection with the etiology of this form of anæmia there is evidence of a double infective process: (1) a specific infection of which the chief evidence is a glossitis; (2) a septic infection of mouth, stomach, and intestine of which the chief evidences during life are varying degrees of "oral sepsis" and "septic gastritis," the latter recognisable during life by the vomit and various symptoms of oral gastric and intestinal disturbance, and after death by the conditions of gastritis, gastric atrophy, intestinal atrophy, etc., now and again found in the disease. Dr. Hunter declines to admit what is asserted by various German observers, namely, that in pernicious anæmia we have merely to do with a frequently occurring group of symptoms met with in very different conditions of disease. His conclusion is that in it we have to do with a disease *sui generis*—a definite, specific, infective disease, with a very definite group of clinical features and a very definite and constant group of pathological changes associated with it, these changes being of two kinds: (1) infective lesions in the mucosa of the alimentary tract; (2) distinctive pigment changes in the liver and kidneys, the result of the hæmolysis caused by the infection underlying the disease. The next step must be the isolation of the infection by which the specific glossitis is set up.

